# Key-Marker Volatile Compounds in Aromatic Rice (*Oryza sativa*) Grains: An HS-SPME Extraction Method Combined with GC×GC-TOFMS

**DOI:** 10.3390/molecules24224180

**Published:** 2019-11-18

**Authors:** Widiastuti Setyaningsih, Tomasz Majchrzak, Tomasz Dymerski, Jacek Namieśnik, Miguel Palma

**Affiliations:** 1 Department of Food and Agricultural Product Technology, Faculty of Agricultural Technology, Gadjah Mada University, Jalan Flora No. 1, Bulaksumur, Depok, Sleman, Yogyakarta 55281, Indonesia; widiastuti.setyaningsih@ugm.ac.id; 2 Department of Analytical Chemistry, Faculty of Chemistry, Gdańsk University of Technology, Narutowicza 11/12 Str., 80-233 Gdańsk, Poland; tomasz.majchrzak1@pg.edu.pl (T.M.); tomasz.dymerski@pg.edu.pl (T.D.); chemanal@pg.edu.pl (J.N.); 3 Department of Analytical Chemistry, Faculty of Sciences, IVAGRO, Campus del Rio San Pedro, University of Cadiz, Puerto Real, 11510 Cadiz, Spain

**Keywords:** aromatic rice, fractional factorial design, multi-response optimisation, volatile compounds, principal component analysis

## Abstract

The aroma of rice essentially contributes to the quality of rice grains. For some varieties, their aroma properties really drive consumer preferences. In this paper, using a dynamic headspace solid-phase microextraction (HS-SPME) system coupled to a two-dimensional gas chromatography (GC×GC) using a time-of-flight mass spectrometric detector (TOFMS) and multivariate analysis, the volatile compounds of aromatic and non-aromatic rice grains were contrasted to define some chemical markers. Fifty-one volatile compounds were selected for principal component analysis resulting in eight key-marker volatile compounds (i.e., pentanal, hexanal, 2-pentyl-furan, 2,4-nonadienal, pyridine, 1-octen-3-ol and (*E*)-2-octenal) as responsible for the differences between aromatic and non-aromatic rice varieties. The factors that are most likely to affect the HS-SPME efficiency for the aforementioned key-marker compounds were evaluated using a $2_{III}^{5-2}$ fractional factorial design in conjunction with multi-response optimisation. The method precision values, expressed as % of coefficient of variation (CV), were ranging from 1.91% to 26.90% for repeatability (*n* = 9) and 7.32% to 37.36% for intermediate precision (*n* = 3 × 3). Furthermore, the method was successfully applied to evaluate the volatile compounds of rice varieties from some Asian countries.

## 1. Introduction

Indonesia is the world’s third-largest rice producer in addition to one of the world’s major rice consumers [1]. Within this region, rice dominates not only food security but also national economies. Rice has been cultivated in Indonesia from the time between 2000 and 1400 B.C., while the production has considerably increased since 1925, thereby giving rise to a number of rice varieties. There are two groups of the grains based on their aroma (i.e., aromatic and non-aromatic) [2].

Some rice varieties are known as aromatic rice. They contain some typical volatile compounds released from the grain that discriminate these rice varieties from the ordinary ones [3]. These varieties have become more widely appreciated in the current market for their specific aroma properties in addition to their appearance and taste. Since the grain aroma is a primary sensory attribute of high-quality rice that has a critical impact on consumer preference, recent researches have led to an increase in rice breeding programs and genetic modifications focusing on the odour profile to generate high-quality aromatic rice cultivars [4]. Henceforth, an analytical method for key-marker volatile compounds determination is crucial to facilitate the characterization [5] that is useful for the selection of lines with superior quality attributes.

In addition to the marker-assisted breeding in question, the need for a novel analytical method to improve the accuracy of the determination of volatile compounds is also essential to confirm the geographical origin discrimination [6]. Therefore, in particular, this study comprised three main parts: (i) Contrast the volatile composition of Indonesian aromatic and non-aromatic rice varieties to define the key-marker volatile compounds; (ii) focus on optimisation and validation of the analytical method for the extraction of key-marker compounds from rice grains; and, lastly, (iii) applying the developed method to assess a number of aromatic rice samples.

Research into key-marker volatile compounds in rice was started more than thirty years ago [7] and this has continued to be an active field of the recent studies, indicated by numerous reports mainly focused on a single compound recognised as the most important marker for rice volatile, viz., 2-acetyl-1-pyrroline [8,9,10,11,12,13]. However, updated researches on volatile compounds that contribute to prominent distinction of a premium quality of rice grains have been limited by the concentration of the compounds and complexity of rice matrices that contain a diverse range of primary and secondary metabolites [14].

A two-dimensional gas chromatography (GC×GC) coupled with time-of-flight mass spectrometry (TOFMS) detector offers a solution to the aforementioned problem, as a cutting-edge chromatographic technique that provides complete separation and full scan collection of spectral data, for thousands of compounds to low pg Kg^−1^ concentrations. This approach can provide a broad fingerprint, which greatly increases the probability of recognising new compounds and commences potential key-marker volatile compounds. In this study, volatile compounds identified by the GC×GC-TOFMS were then evaluated using principal component analysis (PCA) for screening the main compounds that are responsible for the typical volatile compounds of aromatic rice.

Prior to GC×GC-TOFMS analysis, modern studies have shown that headspace solid-phase microextraction (HS-SPME) is a suitable sample preparation technique to increase the extraction efficiency for various trace compounds in food matrices [15,16,17]. The factors that influence the yield of the HS-SPME are predominantly related to adsorption time and temperature. Additionally, pre-incubation time and headspace volume were also found to affect the HS-SPME recovery [18]. As a number of factors can involve in the course of the extraction, the screening and optimisation of the significant factors must be carried out in order to establish a reliable analytical HS-SPME method.

In the study described here, five extraction factors were evaluated (i.e., pre-incubation time, adsorption time, adsorption temperature, the amount of rice sample and added water). A factorial design with a reduced number of runs can provide enough information to reach reliable results. This option is specifically interesting if more than four factors are going to be evaluated. Therefore, a chemometric approach based on a fractional factorial design (FFD) is a reasonable option to evaluate the significance of the studied factors prior to optimising the HS-SPME conditions [19] and has been used in this study.

The option of defining the optimised conditions for an extraction becomes more difficult when the total recovery is described for a multi-compound extraction. It is typically important to find a compromise among conflicting goals for compounds that respond differently to significant factors of extraction. Therefore, the optimisation of a multi-compound extraction necessitates criteria that allow the simultaneous optimisation (i.e., multi-response optimisation (MRO)) approach.

The desirability function has become an increasingly popular practice for multi-response optimisation. Individual response surfaces are determined for each response of MRO. This function has been successfully used for the optimisation of analytical systems, which involve several responses. Henceforth, to achieve the aforementioned objective of developing the optimised HS-SMPE conditions for key-marker volatile compounds in rice, FFD in conjunction with MRO was used in this study. 

## 2. Results and Discussion

### 2.1. Volatile Compounds from Indonesian Rice

A number of Indonesian rice samples, including non-aromatic (IR64, C4 Raja and C4 Dewi Sri) and aromatic (Rojolele, Pandan Wangi and Mentik Wangi) varieties, were studied by the use of GC×GC-TOFMS. The results of this chromatographic analysis for volatiles in six Indonesian rice varieties are listed in Table 1. More than a hundred volatiles were identified and most of these have been found in rice grains previously [12,20,21,22,23,24,25,26,27,28,29,30,31,32,33,34,35,36]; while several compounds, such as 1,3-octadiene, 1-octen-3-yl acetate, isomenthol, estragole, and trans-anethole, were identified in rice samples for the first time.

The essential objective of this particular research is the identification of marker compounds, indicating the existence of quality features sought after for the studied rice samples. Therefore, among the volatiles identified by GC×GC-TOFMS, fifty-one odour-active compounds were selected since the compounds were known to contribute to the unique flavour of a cross-section of rice cultivars [27,37,38,39,40], besides having a variability of the levels in the tested rice samples. These compounds were then further studied.

PCA was performed on the data of odour-active compounds concentration in aromatic and non-aromatic rice varieties, to assess the possibility of defining the key-marker compounds in aromatic grains. From the analysis, five components were extracted due to having eigenvalues ≥ 1.0 that account for 99.99% of the variability in the original data.

**Figure 1:**
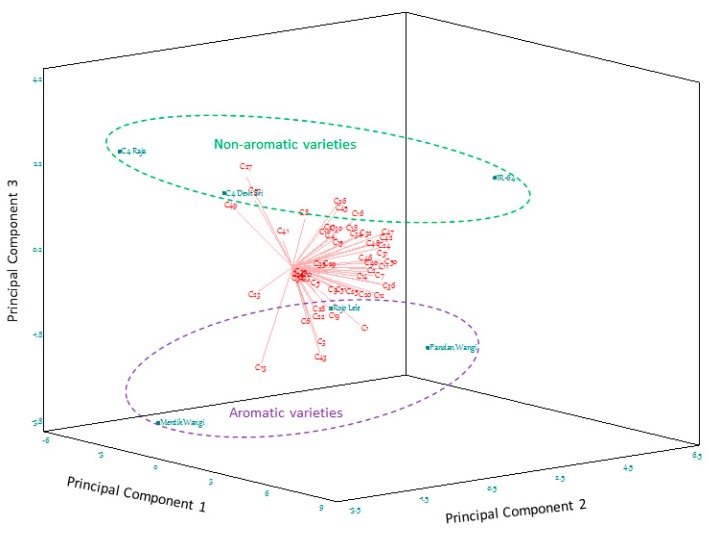
PCA 3D biplot for aromatic (Rojolele, Pandan Wangi and Mentik Wangi) and non-aromatic (IR64, C4 Raja and C4 Dewi Sri) rice samples and the variables used. Fifty-one volatile compounds were used as variables in the PCA (see Table 1).

Meant for appropriate assessment of the regression analysis, a biplot of correlation loadings is preferable to conventional loading plots, as it provides easier interpretation of the relationships between volatile compounds and rice varieties (Figure 1). The technique described here permits an effective tool to define the key-marker compounds of Indonesian aromatic rice varieties.

The PCA 3D biplot accounted for 83.98% of the total variance, with principal component 1 (PC1), PC2, and PC3 explaining 48.07%, 24.09% and 11.82%, respectively. The six rice varieties were alienated revealing the probability of distinctive volatile compounds profiles (Figure 1). The group of non-aromatic rice varieties was plotted on the positive axis of PC3, while aromatic varieties were laid on the opposite coordinate along the PC3 axis.

The scent of both aromatic and non-aromatic rice involved the combination of odour-active compounds [23,31]. In aromatic rice, two compounds in negative PC3, 2-acetyl-1-pyrroline (C13) and 2,4-Nonadienal (C43), were considered remarkably essential. Particularly, 2-acetyl-1-pyrroline occurred in relatively lower concentration compared with other volatile compounds, but it is presented in aromatic rice varieties with different levels.

Eucalyptol (C27), linalool (C37), and 1H-indole (C49) were much more dominant in non-aromatic than in aromatic cultivars (positive PC3). The relative content of linalool (C37) has been reported to be increased with drought stress [41] as a result of quality improvement for some non-aromatic cultivars. The compounds with a value near zero in PC3, such as 2-butylfuran (C10), guaiacol (C33), o-cymene (C38) and trans-2-nonenal (C39), did not produce clear distinctions between aromatic and non-aromatic rice varieties due to the similar levels of these compounds in the grains.

Nonetheless, in regards to the PC2 axis, the non-aromatic rice varieties can be separated. IR64 (positive PC2) can be noticeably discriminated to the C4 varieties (negative PC2). The non-aromatic rice samples studied here were developed in a major advance in rice production, as it provided higher yield potential for their specific land assignments. IR64, also known as Sentra Ramos, is the most common rice in the Indonesian market attributable to its massive production within the region. In contrary, C4 Raja and C4 Dewi Sri are only produced in extreme land, as the plant was designed to adapt to the heat and drought in some regions [42]. This fact may explain the distinctive aroma profile of these varieties with the other non-aromatic variety, viz., IR64.

Likewise, PC2 also distinguished the rice within the aromatic group. Additionally, specific volatile compounds characterised specific aromatic rice varieties. Mentik Wangi was principally explained by 2-acetyl-1-pyrroline (C13), while pentanal (C1) largely described Pandan Wangi. In contrast, Rojolele is depicted by more than one volatile compound and emanates a stronger aroma than other aromatic rice. It is; therefore, recognised as an elite grain in the Indonesian rice market.

In addition to being considered as aromatic rice, together with Mentik Wangi, Pandan Wangi is described as a round-shaped and relatively thick grain [43]. Rojolele rice is characterised by long slender grains with a high elongation ratio. The differences in physical characteristics endorse some expectations of discrepancies in chemical markers.

Based on these results, volatile compounds most directly related to PC3 were considered as the typical volatile compounds for aromatic rice varieties. These critical volatile compounds account for differences among aromatic and non-aromatic rice varieties. Hence, eight volatile compounds: pentanal (C1), pyridine (C3), hexanal (C6), 2-acetyl-1-pyrroline (C13), 1-octen-3-ol (C19), 2-pentylfuran (C22), (*E*)-2-octenal (C28) and 2,4-nonadienal (C43) were defined as the key-markers of volatile compounds separating aromatic and non-aromatic rice varieties. Subsequently, a reliable analytical method using HS-SPME for these compounds in rice was developed in this study.

### 2.2. Optimisation of HS-SPME for the Key-Markers in Aromatic Rice

The variables that were likely to influence the extraction of key-marker compounds from aromatic rice were optimised. The factors considered were the amount of the sample (*x*_1_), the volume of water (*x*_2_), adsorption temperature (*x*_3_), pre-incubation time (*x*_4_), and adsorption time (*x*_5_). Based on the experimental design generated by the $2_{III}^{5-2}$ FFD with two centre points, 11 extraction processes were completed to extract the key-marker compounds from rice (Table 2).

The response for each extraction in the experimental design generated by the $2_{III}^{5-2}$ FFD was calculated and expressed as the value relative to the maximum yield obtained (%) for the individual level of key-marker aroma compounds in rice (i.e., pentanal (C1), pyridine (C3), hexanal (C6), 2-acetyl-1-pyrroline (C13), 1-octen-3-ol (C19), 2-pentylfuran (C22), (*E*)-2-octenal (C28) and 2,4-nonadienal (C43)). The responses were simultaneously optimized using MRO, wherein the optimization target for each response was considered equivalently important. The importance of the responses for computational analysis was indicated by the impact coefficient given to the responses in the MRO. By default, values of the impact coefficients were set to three (STATGRAPHICS Centurion XVI, Warrenton, VA, USA) with medium sensitivity.

Prior to MRO, the response surface methodology (RSM) data were formerly analysed to generate a model for each response separately. The efficiency of the model was checked by ANOVA and the suitability of the model was judged by considering coefficient of determination (*R*^2^). The values of the *R*^2^ statistic ranged from 68.05% (2PF) to 95.96% (OCA). Henceforth, the RSM for each response was confirmed to provide a high degree of correlation between the experimental and predicted values.

As the response surface equation constructed by the software for each response was plotted, the model provides the variable effects on the response over the studied range of the $2_{III}^{5-2}$ FFD. Subsequently, the desirability function d(y) was then constructed based on the values obtained for each optimized response. The MRO approach assumes the response values equal to (y) can be modelled through the d(y), where the desirability ranges from di(ŷi) = 0 for an undesirable response and di(ŷi) = 1 represents a completely desirable value. The target optimization defined by MRO was to maximize the HS-SPME recovery (100% extraction yield) of each key-marker aroma compound simultaneously. To obtain these optimum values, the d(y) was plotted as a 3D contour plot, which illustrated the optimum point of the simultaneous optimization (Figure S2 Supplementary Material).

The proposed ordinates and optimal conditions for HS-SPME by MRO were as follows: Amount of the sample (*x*_1_, −1.00, 0.5 g), volume of water (*x*_2_, 1.00, 5 mL), adsorption temperature (*x*_3_, 0.36, 80.73 °C), pre-incubation time (*x*_4_, −1.00, 5 min), and adsorption time (*x*_5_, 1.00, 50 min). Because the value for adsorption time was in the corner of the studied range for this extraction variable, it was decided to study values above the highest assayed level.

The results of extraction yields by different adsorption times are shown in Figure S3 in Supplementary Material. A single-factor ANOVA was used to evaluate the significance of adsorption time in the extraction yield. The adsorption time of 70 min was found to have a significant effect on the extraction yield because the Fcalculated for adsorption time (5.21) was higher than Fcritical (2.84). A longer extraction time results in a decrease of the extracted compounds, attributable to a longer process, and applying relatively high temperature may ruin the stability of the target compounds. As a result, 70 min was defined as the optimum adsorption time.

### 2.3. Method Validation of HS-SPME GC×GC-TOFMS

The analytical procedure for the extraction of volatile compounds was validated according to the recommendations of ISO 17,025 and the International Council for Harmonisation (ICH) Guideline Q2 (R1) [44,45]. Under the optimum experimental conditions, the validation of the proposed HS-SPME GC×GC-TOFMS method involving HS-SPME followed by GC×GC-TOFMS was accomplished.

The precision of the method was evaluated by assessing repeatability (intra-day) and intermediate precision (extra-day). Precision was expressed as the coefficient of variation (CV). The method precision values, expressed as % CV, of the developed method ranged from 1.91% (2PF) to 26.90% (PYR) for repeatability (*n* = 9), and 7.32% (OCA) to 37.36% (PEN) for intermediate precision (*n* = 3 × 3). The result confirmed that acceptable precision for the extraction method had been achieved.

A certified reference material was not available for the studied compounds in rice matrices; consequently, definitive statements cannot be made with regard to accuracy. Nonetheless, the extraction recovery (%R) was determined after evaluating the results from spiked rice samples with standards. The recoveries related to the spiked standards on rice samples ranged from 78.79% (2PF) to 96.86% (OCT). These results show that the developed extraction method is applicable for the assessment of studied volatile compounds.

### 2.4. Real Rice Samples Application of HS-SPME

**Figure 2:**
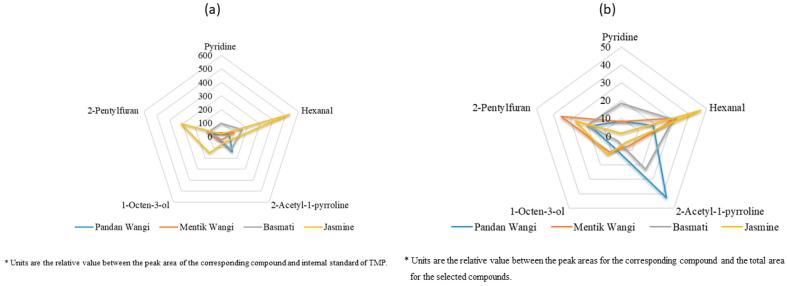
The relative levels of key-marker volatile compounds in tested rice grain samples (**a**) and the proportion of the compounds contributing to the total aroma compounds (**b**).

To evaluate the efficiency of the proposed method in real samples, the developed HS-SPME was applied to assay the key-marker volatile compounds in several aromatic rice samples, including aromatic rice from Indonesia (Pandan Wangi and Mentik Wangi), India (Basmati) and Thailand (Jasmine). Volatile profiles were obtained from these samples, then compared in order to establish differences. The results of real sample application experiments is shown in Figure 2.

The four tested rice samples are considered as aromatic rice varieties in the national and international market [46,47]. Pandan wangi and Basmati had the highest proportion of 2-acetyl-1pyrroline, whilst hexanal and 2-pentylfuran were the most prominent volatile compounds for Jasmine and Mentik Wangi. The different levels of key-marker volatile compounds in aromatic rice samples could be due to different regions for cultivation [48].

Since 1983, 2-acetyl-1pyrroline is regarded as the solely most important compound in rice, especially fragrant or aromatic rice [7]. However, it was not the case for Kao Dok Mali 105 or the so-called Thai Jasmine rice and Mentik Wangi. Apart from 2-acetyl-1-pyrroline, other key-marker volatile compounds were also counted as important compounds that affect the quality of aromatic rice, including hexanal and 2-pentylfuran. The result also disclosed that Jasmine rice has a markedly higher amount of key-marker compounds compared with other tested aromatic rice samples.

## 3. Materials and Methods

### 3.1. Chemicals and Reagents

Standard compounds of the highest available purity were used. Pentanal (PEN), hexanal (HEX), 2-pentyl-furan (2PF), 2,4-nonadienal (NON), pyridine (PYR), 1-octen-3-ol (OCT), (*E*)-2-octenal (OCA) and 2,4,6-trimethylpyridine (TMP) were purchased from Sigma-Aldrich (St. Louis, MO, USA). Water was purified with a Milli-Q purification system A10 Gradient/Elix System (Millipore, Bedford, MA, USA). A standard stock solution of TMP at 0.1 mg L^−1^ was prepared in Milli-Q water, stored in a sealed vial at 4 °C, and used as internal standard.

### 3.2. Natural Source of 2-Acetyl-1-Pyrroline

There is not a commercially available standard for this compound. Therefore, Pandan (*Pandanus amaryllifolius*) leaf was selected as a natural source of 2-acetyl-1-pyrroline (2AP) as the abundant amount of this compound in the leaves has been previously described [10,13,49]. Fresh Pandan leaves were acquired from a local supplier in Yogyakarta, Indonesia. The leaves were cut into pieces ±1 mm in size and stored in a sealed vial at 4 °C. The identity of 2AP in Pandan leaves was confirmed by HS-SPME GC×GC-TOFMS using the NIST 2011 mass spectral library (Figure S1 Supplementary Material). It was used only for identification purposes.

### 3.3. Rice Grains and Sample Preparation

In the initial study, three non-aromatic rice (IR64, C4 Raja and C4 Dewi Sri) were used to contrast with three aromatic varieties (Rojolele, Mentik Wangi and Pandan Wangi) to define the key-marker volatile compounds in grain [46]. The samples used in this study were fully polished grains of the white rice variety. The rice sample (2.5 g) and Milli-Q water (5 mL) was placed in a 15 mL vial, which was then tightly capped with an open top closure with PTFE/silicone septa.

An aromatic rice variety of Pandan Wangi was selected for the study to develop an optimised extraction method of key-marker compounds. Subsequently, the final extraction method was applied to a number of aromatic rice products available in the international market (Basmati and Jasmine) and the Indonesian national market (Rojolele, Pandan Wangi and Mentik Wangi) from a different region of origins in Java Island. Several samples (IR64, C4 Raja, C4 Dewi Sri, Rojolele, Mentik Wangi and Pandan Wangi) were acquired from a smallholder rice distributor in the Central Java area, Indonesia. These samples were harvested no more than 6 months before being used. Some samples (Basmati and Jasmine) were obtained from a commercial market in Spain, no information about the harvest period was found about these samples. A rice sample (20 g) was placed in a plastic cylinder and the rice grains were milled with an Ultraturrax homogenizer (IKA^®^ T25 Digital, Staufen, Germany) for 10 min prior to extraction. Every 1 min, the milling process was stopped to avoid excessive heating of the sample. The fine powder of rice grain was then homogenized by stirring and the sample was stored in a closed container in a refrigerator before being used for analysis. Samples were analysed over a period of two weeks.

### 3.4. Headspace Solid-Phase Microextraction (HS-SPME)

Volatile compounds from the rice samples were extracted using a dynamic headspace solid-phase microextraction (HS-SPME) attached with divinylbenzene/carboxen/polydimethylsiloxane (DVB/CAR/PDMS) StableFlex fibre of 50/30 μm thickness and 2 cm length (Sigma-Aldrich, Saint Louis, MO, USA). According to the experimental design, rice grains were accurately weighed at either 0.5, 1.5 or 2.5 g and Milli-Q water was loaded at either 0, 2.5 or 5.0 mL into a 15 mL screw top vial, then 100 µL of aqueous solution containing 5 ng of 2,4,6-trimethylpyridine (TMP) (Sigma-Aldrich, Saint Louis, MO, USA) as the internal standard was added and the vial was sealed with PTFE/silicone septa. The HS-SPME was carried out according to the design of experiment (DOE), varying the extraction factors of equilibration time (5–15 min), adsorption temperature (40–100 °C) and adsorption time (10–50 min). Thermal desorption of the analytes from the SPME fibre was done at 250 °C. Before starting the extraction, 0.1 mL of TMP standard solution was added into the sample. Every peak area in the chromatograms were standardized by the resulting area for the TMP peak.

### 3.5. GC×GC-TOFMS Analysis

Analysis was performed using a Pegasus 4D GC×GC instrument (LECO, St. Joseph, MI, USA), including an Agilent 6890A GC (Agilent Technologies, Palo Alto, CA, USA) coupled with Pegasus IV time-of-flight mass spectrometer (LECO Corp., St. Joseph, MI, USA) and Gerstel MPS2 auto-sampler (Gerstel, Mülheim, Germany). The column set consisted of a 30 m × 0.25 mm × 0.25 μm primary column (1D) with Equity 1 stationary phase (Supelco, Bellefonte, PA, USA) and a 2.0 m × 0.10 mm × 0.10 μm secondary column (2D) with Sol–Gel–Wax stationary phase (SGE Analytical Science, Austin, TX, USA). A modulation period of 5.0 s was used with the cryogenic trap cooled to −196 °C by liquid nitrogen.

The volatile compounds were separated using the following temperature gradient program for the primary GC oven: Initial temperature of 40 °C maintained for 1 min, then ramped at 8 °C/min to 250 °C, and finally kept for 10 min. The temperature program for the secondary GC oven was with the shift of +40 °C according to the program of primary GC oven. The total analysis time was 37 min. The injector was carried out in splitless mode at 250 °C. Helium was used as the carrier gas at a constant flow of 1.0 mL/min. The temperatures for the transfer line and ion source were maintained at 250 °C. The detector voltage was set to 1600 V. Ions in the *m*/*z* 40–500 range were analysed with a data acquisition rate of 125 spectra/s.

### 3.6. Experimental Design and Optimisation

The effect of the tested independent factors on the response within the studied range was evaluated by performing a fractional factorial design (FFD) (i.e., a $2^{5-2}$ (quarter fraction) with two central points of analysis). The extraction factors included in the design were amount of the sample (*x*^1^, 0.5–2.5 g), volume of water (*x*_2_, 0–5 mL), adsorption temperature (*x*_3_, 40–100 °C), pre-incubation time (*x*_4_, 5–15 min), and adsorption time (*x*_5_, 10–50 min). Since the variables have different units and ranges, each of the variables was first normalised and forced to range from −1 to +1 in order to obtain a more even response. Therefore, the factor levels were denoted as −1 (low), 0 (central point) and +1 (high) according to the following equation:

(1) $$x_{i}=\frac{x_{i}-x_{0}}{∆x},$$

where *x*_i_ is the coded value of the factor *x*_i_, *x*_0_ is the value of *x* at the centre point, and Δ*x* is the increment of *x*_i_ corresponding to a variation per unit of *x*_i_. The factors included in the design are shown in Table 3 along with their respective levels.

The design of experiment (DOE) matrix was established with resolution (R) of III, wherein every main effect is confounded (aliased) with at least one first-order interaction. The $2_{III}^{5-2}$ fractional factorial design allowed the first three variables (*x*_1_ to *x*_3_) to be set and thus the DOE was obtained by establishing the full $2^{3}$ factorials as the basic design (with the three factors *x*_1_, *x*_2_ and *x*_3_) and factors *x*_4_ and *x*_5_ were subsequently equated to the *x*_1_*x*_2_ and *x*_1_*x*_3_ interactions, respectively. This particular design produced the following defining relationships: I = *x*_1_ *x*_2_ *x*_4_ = *x*_1_ *x*_3_ *x*_5_ = *x*_2_ *x*_3_ *x*_4_ *x*_5_. The linear model for this fractional factorial design is:

(2) $$y=\beta_{0}+\overset{k}{\underset{i=1}{\sum }}\beta_{i}x_{i}+\sum \sum_{j<i}\beta_{\mathrm{ij}}x_{i}x_{j}+\varepsilon ,$$

where *β*_i_ (i = 1, 2, ..., 5) is the parameter estimated for the factor i, *β*_ij_ (i = 1, 2, ..., 5; j = 1, 2, ..., 5) is the parameter estimated for the interaction between variables i and j; *x*_i_ is the coded form of factor i that influences the response *y*; and *x*_i_ is the coded form of factor i that influences the response y. The whole design consisted of 11 runs carried out in random order and these are presented in Table 3.

Principal component analysis (PCA) and multi-response optimisation (MRO) were performed with the trial version of STATGRAPHICS Centurion XVI (Statpoint Technologies, Inc., Warrenton, VA, USA) to define and optimise the key-marker compounds of aromatic rice grains. The experimental results in single factor experiments were analysed using Gnumeric 1.12.17. The analysis of variance (ANOVA) and least significant difference (LSD) test were used to determine the significance of differences between the means.

## 4. Conclusions

Eight volatile compounds were found as chemical key-markers for different rice grains varieties using HS-SPME GC×GC-TOFMS and chemometric analysis. These compounds were effectively extracted using HS-SPME under the following optimised conditions: Amount of the sample (0.5 g), volume of water (5 mL), adsorption temperature (80.73 °C), pre-incubation time (5 min), and adsorption time (50 min). The validation of HS-SPME ensured acceptable precision and accuracy of the method. In addition, the method developed based on HS-SPME GC×GC-TOFMS was successfully applied to evaluate the volatile compounds of four aromatic rice varieties, thus considered as a reliable analytical method for the key-marker compounds in rice grains.

## Figures and Tables

**Table 1:** GC×GC-TOFMS analysis for volatiles in six Indonesian rice varieties.

No	Compounds	Retention Time (s)		Mass ^3^	References	Odour Strength ^4^	Odour Description ^4^
		1D ^1^	2D ^2^				
**1**	**Pentanal ^C1^**	**435**	**2.11**	**58**	[20,21,22,23,24,25]	**High, 1%**	**Bready; fruity; nutty; berry**
2	Acetic acid	455	4.41	60	[21,22,26]	High, 10%	Pungent; sour; vinegar
3	2-Methylfuran ^C2^	495	2.59	53	[22]	Medium, 1%	Ethereal; acetone; chocolate
**4**	**Pyridine ^C3^**	**505**	**2.60**	**79**	[21,23,25,27]	**Very high, 0.01%**	**Sour; putrid; fishy; amine**
5	1-Pentanol ^C4^	520	2.47	42	[12,21,23,25]	High, 10%	Pungent; fermented; bread; yeasty; fusel; winey
6	Toluene ^C5^	525	2.14	91	[21,22,23]		Sweet
7	2-Hexanone	540	2.16	43	[23,28]	High, 1%	Fruity; fungal; meaty; buttery
**8**	**Hexanal ^C6^**	**550**	**2.17**	**56**	[12,20,21,22,23,24,25,26,27,29,30,31]	**High, 1%**	**Fresh; green; fatty; grass; leafy; fruity; sweaty**
9	Furfural ^C7^	585	3.38	95	[23,25,26,27,32,33]	Medium, 1%	Sweet; woody; almond; fragrant baked; bread
10	2-Methylpyridine	590	2.45	93	[23,27]		Astringent; hazelnut
11	1,3-Octadiene *	600	1.99	54			
12	1-Hexanol ^C8^	645	2.45	56	[12,23,29]	Medium, 10%	Pungent; ethereal; fusel; oil; fruity; alcoholic; sweet with a green top note
13	1,3-Dimethylbenzene	655	2.14	91	[12,23,29]		Fried; medicine; nut; plastic; rancid
14	2-Heptanone ^C9^	665	2.18	58	[20,21,22,23,26,27,29]	High, 10%	Cheesy; fruity; spicy; sweet; herbal; coconut; woody
15	2-Butylfuran ^C10^	680	2.09	81	[21,23,29,30]	Medium	Mild; fruity; wine; sweet; spicy
16	Heptanal ^C11^	680	2.17	70	[20,21,22,23,24,25,26,27,29]	High, 1%	Fresh; fatty; green; herbal
17	Styrene	680	2.30	104	[21,26,27]		
18	1,2-Dimethylbenzene ^C12^	685	2.18	91	[12,21]		Geranium
**19**	**2-Acetyl-1-pyrroline ^C13^**	**700**	**2.45**	**43**	[12,20,21,23,24,25,30,31,34]	**Medium, 1%**	**Popcorn; toasted; grain; malty**
20	2-Methyl-5-isopropenylfuran ^C14^	730	2.18	122	[22]	Medium	Sweet; spearmint; herbal
21	(Z)-2-heptenal ^C15^	750	2.32	41	[20,22,23,29]	High, 1%	Pungent; green; vegetable; fresh; fatty
22	Benzaldehyde ^C16^	750	2.98	105	[20,21,23,25,26,27,29,30]	High, 10%	Bitter; almond; burnt sugar; cherry; malt; roasted pepper
23	1-Ethyl-4-methylbenzene	775	2.14	105	[21,23]		
24	1-Heptanol ^C17^	775	2.39	70	[12,22,27,30]	Medium, 10%	Musty; leafy; herbal; green; sweet; woody
25	Benzonitrile	775	3.27	103	[29]		
**26**	**1-Octen-3-ol ^C19^**	**785**	**2.43**	**57**	[20,22,23,24,25,27,30,31]	**High, 10%**	**Earthy; green; oily; vegetative; fungal**
27	6-Methyl-5-hepten-2-one ^C18^	785	2.26	43	[20,21,23,24,25]	Medium, 10%	Citrus; green; musty; lemongrass
28	Phenol ^C20^	790	2.99	66	[12,23,26,29]	High, 0.01%	Phenolic
29	2-Octanone ^C21^	795	2.18	58	[22,23,25]	Med, 10%	Earthy; weedy; natural; woody
30	2,4,6-Trimethylpyridine **	800	2.30	121			
**31**	**2-Pentylfuran ^C22^**	**805**	**2.09**	**81**	[20,21,22,23,24,27,29,30]	**High, 10%**	**Fruity, green, earthy beany with vegetable-like nuances**
32	α-Myrcene ^C23^	810	2.02	93	[35]	Med, 5%	Peppery; terpene; spicy; balsam
33	Octanal ^C24^	810	2.17	43	[20,21,22,23,24,25,27,29,30,31]	High, 1%	Waxy; citrus; orange peel; fatty
34	1,2,3-Trimethylbenzene	815	2.18	105	[22,23]		Pesticide
35	α-Phellandrene	835	2.03	93	[29]	Med, 5%	Citrus; herbal; terpene; green; woody; peppery
36	1,2,4-Trimethyl benzene	850	2.22	105	[23,30]		pesticide, plastic
37	1-Nitro-hexane	850	2.49	43	[30]		
38	3-Octen-2-one ^C25^	850	2.30	55	[21,22,23,27]	High, 1%	Earthy; spicy; herbal; sweet; mushroom
39	Benzene acetaldehyde ^C26^	850	3.00	91	[21,22,25,27]	High, 2%	Honey; floral; rose; sweet; cocoa
40	1-Ethyl-2-methylbenzene	860	2.26	105	[22]		
41	Isobutyl nonyl ester oxalic acid	860	2.16	57	[24]		
42	Eucalyptol ^C27^	865	2.06	43	[20,27]	High, 10%	Eucalyptus; herbal; camphor
**43**	**(E)-2-octenal ^C28^**	**875**	**2.30**	**70**	[20,21,22,23,24,25,26,27,29,30]	**High, 1%**	**Fresh; cucumber; fatty; green; herbal**
44	Indene	875	2.46	115	[26]		
45	(Z)-3,7-Dimethyl-1,3,6-octatriene ^C29^	880	2.05	93	[26]	Medium	Tropical; green; woody with vegetable nuances
46	1-Phenyl-ethanone ^C30^	885	2.93	105	[25,27,29]		
47	Dihydromyrcenol	900	2.26	59			
48	1-Octanol ^C31^	900	2.33	56	[20,21,22,23,25,27,30,31]	Medium 10%	Waxy; green; orange; rose; mushroom
49	Decane	910	2.11	57	[21,22,23,30]		Alkane; odour
50	3,5-Octadien-2-one ^C32^	915	2.52	95	[34]	High, 1%	Fruity; fatty; mushroom
51	Guaiacol ^C33^	915	3.57	109	[32,36]	High, 1%	Phenolic; smoke; spice; vanilla woody
52	2-Nonanone ^C34^	920	2.17	58	[20,22,23]	Medium	Fresh; sweet; green; weedy earthy herbal
53	6-Methyl-3,5-heptadiene-2-one ^C35^	930	2.54	109	[22,25]		Citrus; fruits
54	1-octen-3-yl acetate *	940	2.08	43		Medium	Fresh; green; herbal; lavender; fruity oily
55	α-Terpinolene	935	2.06	93	[35]	Medium, 1%	Sweet; fresh; piney; citrus with a woody old lemon peel nuance
56	Nonanal ^C36^	935	2.17	57	[12,20,21,22,23,25,26,27,30]	High, 1%	Aldehydic; citrus; cucumber fattiness
57	Linalool ^C37^	935	2.33	93	[23,27]	Medium	Citrus; floral; sweet; woody; green; blueberry
58	Decane	940	2.08	57	[22,23,26,30]		
59	Tridecane	950	1.93	43	[23,29,30]		
60	Tetradecane	955	1.92	57	[12,21,22,23,29,30]		
61	o-Cymene ^C38^	970	2.15	119	[29]		
62	(E)-2-nonenal ^C39^	995	2.30	55	[20,21,23,24,25,27,30,31]	High, 1%	Fatty; green cucumber; citrus
63	1,2,3,4-tetramethyl-benzene	1005	2.24	119	[21,23]		
64	2-Decen-1-ol ^C40^	1010	2.14	82	[24]	Medium	Waxy; fresh air; citrus
65	2-Pentylthiophene	1010	2.17	97		High 0.1%	Fruity; fatty; cranberry
66	Ethyl ester benzoic acid ^C41^	1010	2.53	105	[25]	Medium	Fruity; dry musty; sweet; wintergreen
67	1-Nonanol	1015	2.30	56	[12,22,23,27,30]	Medium	Fresh; fatty; floral; rose; orange; dusty; wet; oily
68	Undecane	1025	1.94	43	[21,22,23,29]		Alkane odour
69	(+)-Isomenthol *	1030	2.38	71		Medium, 10%	Mentholic; musty; woody
70	2-Decanone	1035	2.17	58	[23,27]	Medium	Orange; floral; fatty; peach
71	Ethyl ester octanoic acid ^C42^	1040	2.09	88	[21]	Medium	Fruity; wine; waxy; sweet; apricot banana; brandy; pear
72	Naphthalene	1040	2.73	128	[21,23,24,26,27,29,30,34]		Naphthalene
73	Estragole *	1045	2.44	148		Medium	Sweet; sassafrass; anise spice; green herbal; fennel
74	Decanal ^C43^	1050	2.17	57	[20,21,23,25,26,27,29,30,31]	High, 1%	Sweet, aldehydic, orange, waxy and citrus rind
**75**	**2,4-Nonadienal ^C44^**	**1055**	**2.50**	**81**	[21,22,23,30,31]	**High, 0.1%**	**Fatty; green cucumber**
76	Dodecane ^C45^	1065	1.94	43	[21,22,23,30]		Alkane odour
77	Benzothiazole ^C46^	1075	3.36	69	[21,23,24,34]	High, 0.1%	Sulphur-like; rubbery; vegetable; Cooked; nutty; coffee; meat
78	(Z)-2-decenal ^C47^	1110	2.28	70	[22,23,31]	High, 0.1%	Waxy; fatty; earthy; green; mushroom
79	Citral ^C48^	1115	2.41	69	[22,23]	Medium	Sharp lemon; sweet
80	Ethyl ester decanoic acid	1115	2.22	88	[21]	Medium	Sweet; waxy; fruity; apple; grape; oily; brandy
81	Nonanoic acid	1115	3.90	73	[23,25,26]	Medium, 10%	Waxy; dirty; cheese cultured; dairy
82	1-Decanol	1130	2.02	83	[23,24]	Medium	Fatty; waxy; floral; orange sweet; clean watery
83	Trans-anethole *	1140	2.57	148		High, 10%	Sweet anise; liquorice
84	1H-indole ^C49^	1145	1.54	117	[21,22,24,30]	High, 1%	Pungent; floral; animalic; musty; character
85	2-Butyl-1-octanol	1160	1.95	57	[22]		Solvents
86	Undecanal ^C50^	1160	2.14	82	[21,23]	High, 1%	Waxy; soapy; floral; aldehydic; citrus; green; fatty
87	2,4-Decadienal ^C51^	1165	2.46	81	[23,25,26,27,30,31]	High, 1%	Orange; sweet; fresh; citrus fatty; green
88	2,6-Dimethyl-heptadecane	1180	1.95	57	[22]		
89	Dihydro-5-pentyl-2(3H)-furanone	1200	2.95	85	[21,26]	Medium	Creamy; oily with fatty nuances
90	Decanoic acid	1215	3.74	60	[25,26]	Medium, 1%	Unpleasant rancid; sour; fatty; citrus
91	E-2-undecenal	1215	2.27	70	[23,26,30]	High, 1%	Fresh fruity; citrus; orange peel
92	Pentadecane	1220	1.94	57	[21,22,23]		
93	Geranyl acetate	1230	2.22	69	[20,23,31]	Medium, 5%	Floral; rosy; waxy; herbal and green with a slight cooling nuance
94	Hexadecane	1365	1.94	57	[21,22,23,30]		Alkane; root
95	Biphenyl	1240	2.72	154	[30]	High, 0.1%	Pungent; rose; green; geranium
96	1-ethyl-naphthalene	1260	2.60	156	[30]		fatty; earthy
97	Dodecanal	1260	2.16	57	[26,31]	High, 10%	Soapy; waxy; aldehydic; citrus; green; floral
98	(E)-6,10-dimethyl-5,9-undecadien-2-one	1300	2.26	43	[21,25,30]	Medium	Fresh; rose; leaf; floral; green; magnolia; aldehydic
99	1,3-dimethyl-naphthalene	1305	2.71	141	[30]		
100	Trans-caryophyllene	1305	2.08	93	[21]	Medium	Spicy; woody and terpenic
101	α-Ionone	1340	2.36	177	[31]	Medium, 10%	Floral; woody; sweet; fruity; berry; tropical; beeswax
102	2,4-Bis(1,1-dimethylethyl)-phenol	1355	2.95	191	[24]		Phenolic
103	Methyl ester dodecanoic acid	1365	2.123	74	[21]	Medium	Waxy; soapy; creamy; coconut; mushroom
104	α-Copaene	1370	2.11	105	[35]	Medium	Woody; spicy; honey
105	Lilyall	1370	2.46	189	[30]	Medium	Floral; muguet; watery; green; powdery; cumin
106	1S,cis-calamenene	1385	2.19	159	[21]		
107	2,3,6-Trimethyl-naphthalene	1405	2.65	155	[30]		Fruity; dry
108	2-Undecanone	1515	2.18	58	[20,21,22,23]		Waxy; fruity; creamy; fatty; floral
109	Methyl ester decanoic acid	1535	2.14	74	[21]	Medium	Oily; wine; fruity; floral
110	2,6-Diisopropylnaphthalene	1555	2.42	197	[21]		
111	Tetradecanoic acid	1560	3.22	60	[25]	Low, 10%	Faint; waxy and fatty with a hint of pineapple and citrus peel
112	2-Ethylhexyl salicylate	1610	2.39	120	[30]	Low	Mild; orchid; sweet; balsam
113	6,10,14-Trimethyl-2-pentadecanone	1630	2.16	58	[22]	Low	Oily; herbal; jasmine; celery; woody
114	2-Pentadecanone	1670	2.25	58	[30]	Medium, 10%	Fresh; jasmine; celery
115	Methyl ester hexadecanoic acid	1690	2.22	74	[21]		
116	Hexadecanoic acid	1715	3.67	60	[21,23,30]	Low, 1%	Low heavy waxy with a creamy; candle waxy nuance
117	Hexadecanoic acid, ethyl ester	1740	2.26	88	[25]	Low	mild waxy; fruity; creamy milky balsam
118	1-Hexadecanol	1920	2.26	97	[24]	Low	Waxy; floral
119	Heptacosane	2215	2.45	71	[22]		

* Reported for the first time in rice samples and the identification was confirmed by standard compounds. ** Internal standard. ^1^ 1D refers to one-dimensional gas chromatography (GC) separation (in the first column). ^2^ 2D refers to two-dimensional gas chromatography (GC×GC) separation (in the second column). ^3^ Unique mass spectra. The NIST Mass Spectral Database was used to identify volatile compounds from GC×GC coupled with time-of-flight mass spectrometry (TOFM) analyses. ^4^ www.thegoodscentscompany.com. ^Cn^ Odour-active compounds for principal component analysis PCA (*n* = running number of selected compounds). Key-marker compounds are presented in bold letters.

**Table 2:** Selected factors and their levels.

Factors	−1	0	+1	Unit
*x*_1_, sample mass	0.5	1.5	2.5	g
*x*_2_, water volume	0	2.5	5.0	mL
*x*_3_, adsorption temperature	40	70	100	°C
*x*_4_, pre-incubation time	5	10	15	min
*x*_5_, adsorption time	10	30	50	min

**Table 3:** The $2_{III}^{5-2}$ fractional factorial design for five factors with their observed responses.

DOE	Extraction Variables					Extraction Yield (Relative % to Maximum Yield)							
	*x*_1_	*x*_2_	*x*_3_	*x*_4_	*x*_5_	PEN	HEX	PYR	2AP	2PF	OCT	OCA	NON
1	1	1	1	1	1	22.78	3.58	3.80	18.44	21.70	3.89	16.77	7.11
2	−1	−1	1	1	−1	6.05	1.36	1.66	100.00	11.28	9.80	100.00	100.00
3	−1	−1	−1	1	1	30.78	5.79	6.39	22.01	4.85	13.46	61.75	69.87
4	−1	1	1	−1	−1	21.72	1.63	2.04	62.20	9.55	13.73	59.42	22.31
5	−1	1	−1	−1	1	100.00	100.00	100.00	13.40	100.00	100.00	47.52	42.92
6	1	−1	−1	−1	−1	7.55	1.55	1.71	3.43	0.65	1.38	4.61	3.96
7	0	0	0	0	0	10.99	8.33	8.90	54.26	16.14	25.63	32.39	43.71
8	1	1	−1	1	−1	28.99	11.26	11.63	2.88	15.60	6.99	5.10	3.50
9	1	−1	1	−1	1	3.16	0.59	0.73	63.69	3.99	2.24	17.26	25.84
10	0	0	0	0	0	12.66	13.01	8.85	51.67	14.53	23.84	26.91	41.25
11	0	0	0	0	0	15.25	12.51	11.49	48.62	19.49	30.29	31.88	50.60

Abbreviations: Design of experiment (DOE), pentanal (PEN), hexanal (HEX), pyridine (PYR), 2-acetyl-1-pyrroline (2AP), 2-pentyl-furan (2PF), 1-octen-3-ol (OCT), (*E*)-2-octenal (OCA), and 2,4-nonadienal (NON).

## Supplementary Materials

The following are available online, Figure S1: 2-acetyl-1-pyrroline (1) and internal standard 2,4,6-trimethylpyridine (2) in Pandan Leaf. Figure S2: Response surface plots showing the effects of variables (*x*_1_, sample amount; *x*_5_, adsorption time) on the extraction yield. Figure S3: Relative amount of extracted compounds in different adsorption times.

## References

[1] FAOSTAT Food and Agricultural Commodities Production: Countries by Commodity (Rice, Paddy). http://faostat3.fao.org/browse/rankings/countries_by_commodity/E.

[2] Mishra A., Kumar P., Shamim M., Tiwari K.K., Fatima P., Srivastava D., Singh R., Yadav P. (2019). Genetic diversity and population structure analysis of Asian and African aromatic rice (*Oryza sativa* L.) genotypes. J. Genet..

[3] Ghiasvand A.R., Setkova L., Pawliszyn J. (2007). Determination of flavour profile in Iranian fragrant rice samples using cold-fibre SPME–GC–TOF–MS. Flavour Fragr. J..

[4] Shan Q., Zhang Y., Chen K., Zhang K., Gao C. (2015). Creation of fragrant rice by targeted knockout of the OsBADH2 gene using TALEN technology. Plant Biotechnol. J..

[5] Feng S., Huang M., Crane J.H., Wang Y. (2018). Characterization of key aroma-active compounds in lychee (Litchi chinensis Sonn.). J. food drug Anal..

[6] Lim D.K., Mo C., Lee J.H., Long N.P., Dong Z., Li J., Lim J., Kwon S.W. (2018). The integration of multi-platform MS-based metabolomics and multivariate analysis for the geographical origin discrimination of *Oryza sativa* L.. J. Food Drug Anal..

[7] Buttery R.G., Ling L.C., Juliano B.O., Turnbaugh J.G. (1983). Cooked rice aroma and 2-acetyl-1-pyrroline. J. Agric. Food Chem..

[8] Gay F., Maraval I., Roques S., Gunata Z., Boulanger R., Audebert A., Mestres C. (2010). Effect of salinity on yield and 2-acetyl-1-pyrroline content in the grains of three fragrant rice cultivars (*Oryza sativa* L.) in Camargue (France). F. Crop. Res..

[9] Maraval I., Sen K., Agrebi A., Menut C., Morere A., Boulanger R., Gay F., Mestres C., Gunata Z. (2010). Quantification of 2-acetyl-1-pyrroline in rice by stable isotope dilution assay through headspace solid-phase microextraction coupled to gas chromatography-tandem mass spectrometry. Anal. Chim. Acta.

[10] Yahya F., Fryer P.J., Bakalis S. (2011). The absorption of 2-acetyl-1-pyrroline during cooking of rice (*Oryza sativa* L.) with Pandan (*Pandanus amaryllifolius Roxb.*) leaves). Procedia Food Sci..

[11] Grimm C.C., Bergman C., Delgado J.T., Bryant R. (2001). Screening for 2-acetyl-1-pyrroline in the headspace of rice using SPME/GC-MS. J. Agric. Food Chem..

[12] Mahatheeranont S., Keawsa-ard S., Dumri K. (2001). Quantification of the rice aroma compound, 2-acetyl-1-pyrroline, in uncooked Khao Dawk Mali 105 brown rice. J. Agric. Food Chem..

[13] Laohakunjit N., Kerdchoechuen O. (2006). Aroma enrichment and the change during storage of non-aromatic milled rice coated with extracted natural flavor. Food Chem..

[14] Ghiasvand A., Nasirian A., Koonani S., Nouriasl K. (2017). A platinized stainless steel fiber with in-situ coated polyaniline/polypyrrole/graphene oxide nanocomposite sorbent for headspace solid-phase microextraction of aliphatic aldehydes in rice samples. Biomed. Chromatogr..

[15] Dymerski T., Chmiel T., Mostafa A., Sliwinska M., Wisniewska P., Wardencki W., Namiesnik J., Gorecki T. (2013). Botanical and Geographical Origin Characterization of Polish Honeys by Headspace SPME-GC× GC-TOFMS. Curr. Org. Chem..

[16] Abdulra’uf L.B., Tan G.H. (2015). Chemometric approach to the optimization of HS-SPME/GC–MS for the determination of multiclass pesticide residues in fruits and vegetables. Food Chem..

[17] Lim D.K., Mo C., Lee D.-K., Long N.P., Lim J., Kwon S.W. (2018). Non-destructive profiling of volatile organic compounds using HS-SPME/GC-MS and its application for the geographical discrimination of white rice. J. Food Drug Anal..

[18] Tankiewicz M., Morrison C., Biziuk M. (2013). Application and optimization of headspace solid-phase microextraction (HS-SPME) coupled with gas chromatography-flame-ionization detector (GC-FID) to determine products of the petroleum industry in aqueous samples. Microchem. J..

[19] Bianchin J.N., Nardini G., Merib J., Dias A.N., Martendal E., Carasek E. (2014). Screening of volatile compounds in honey using a new sampling strategy combining multiple extraction temperatures in a single assay by HS-SPME-GC-MS. Food Chem..

[20] Griglione A., Liberto E., Cordero C., Bressanello D., Cagliero C., Rubiolo P., Bicchi C., Sgorbini B. (2015). High-quality Italian rice cultivars: Chemical indices of ageing and aroma quality. Food Chem..

[21] Grimm C.C., Champagne E.T., Ohtsubo K., Marsili R. (2002). Analysis of Volatile Compounds in the Headspace of Rice Using SPME/GC/MS. Flavor, Fragrance, and Odor Analysis.

[22] Lin J.-Y., Fan W., Gao Y.-N., Wu S.-F., Wang S.-X. Study on volatile compounds in rice by HS-SPME and GC-MS. Proceedings of the 10th International Working Conference on Stored Product Protection.

[23] Weber D.J., Rohilla R., Singh U.S., Singh R.K., Singh U.S., Khush G.S. (2000). Chemistry and Biochemistry of Aroma in Scented Rice. Aromatic Rices.

[24] Bryant R.J., McClung A.M. (2011). Volatile profiles of aromatic and non-aromatic rice cultivars using SPME/GC–MS. Food Chem..

[25] Buttery R.G., Turnbaugh J.G., Ling L.C. (1988). Contribution of Volatiles to Rice Aroma. J. Agric. Food Chem..

[26] Piyachaiseth T., Jirapakkul W., Chaiseri S. (2011). Aroma Compounds of Flash-Fried Rice. Nat. Sci..

[27] Cho S., Nuijten E., Shewfelt R.L., Kays S.J. (2014). Aroma chemistry of African *Oryza glaberrima* and *Oryza sativa* rice and their interspecific hybrids. J. Sci. Food Agric..

[28] Nadaf A.B., Wakte K.V., Thengane R.J., Jawali N. (2008). Review on Pandanus amaryllifolius Roxb.: The Plant with Rich Source of Principle Basmati Aroma Compound 2 Acetyl-1-Pyrroline. J. Biotechnol..

[29] Calingacion M., Fang L., Quiatchon-Baeza L., Mumm R., Riedel A., Hall R.D., Fitzgerald M. (2015). Delving deeper into technological innovations to understand differences in rice quality. Rice.

[30] Givianrad M.H. (2012). Characterization and assessment of flavor compounds and some allergens in three Iranian rice cultivars during gelatinization process by HS-SPME/GC-MS. E-Journal Chem..

[31] Mahattanatawee K., Rouseff R.L. (2014). Comparison of aroma active and sulfur volatiles in three fragrant rice cultivars using GC-Olfactometry and GC-PFPD. Food Chem..

[32] Setyaningsih W., Saputro I.E., Palma M., Barroso C.G. (2015). Optimisation and validation of the microwave-assisted extraction of phenolic compounds from rice grains. Food Chem..

[33] Setyaningsih W., Saputro I.E., Palma M., Barroso C.G. (2016). Pressurized liquid extraction of phenolic compounds from rice (*Oryza sativa*) grains. Food Chem..

[34] Lloyd S.W., Grimm C.C. Flavor Profiles of Aromatic and non-Aromatic Rice Varieties. Proceedings of the PITTCON Conference and Expo 2010; Pittsburgh Conference.

[35] Lee G.W., Lee S., Chung M.-S., Jeong Y.S., Chung B.Y. (2014). Rice terpene synthase 20 (OsTPS20) plays an important role in producing terpene volatiles in response to abiotic stresses. Protoplasma.

[36] Mathure S.V., Jawali N., Thengane R.J., Nadaf A.B. (2014). Comparative quantitative analysis of headspace volatiles and their association with *BADH2* marker in non-basmati scented, basmati and non-scented rice (*Oryza sativa* L.) cultivars of India. Food Chem..

[37] Yang D.S., Lee K.-S., Kays S.J. (2010). Characterization and discrimination of premium-quality, waxy, and black-pigmented rice based on odor-active compounds. J. Sci. Food Agric..

[38] Maraval I., Mestres C., Pernin K., Ribeyre F., Boulanger R., Guichard E., Gunata Z. (2008). Odor-active compounds in cooked rice cultivars from Camargue (France) analyzed by GC-O and GC-MS. J. Agric. Food Chem..

[39] Widjaja R., Craske J.D., Wootton M. (1996). Comparative studies on volatile components of non-fragrant and fragrant rices. J. Sci. Food Agric..

[40] Jezussek M., Juliano B.O., Schieberle P. (2002). Comparison of key aroma compounds in cooked brown rice varieties based on aroma extract dilution analyses. J. Agric. Food Chem..

[41] Cao P., Liu C., Liu K. (2007). Aromatic constituents in fresh leaves of Lingtou Dancong tea induced by drought stress. Front. Agric. China.

[42] Karki S., Rizal G., Quick W.P. (2013). Improvement of photosynthesis in rice (*Oryza sativa* L.) by inserting the C_4_ pathway. Rice.

[43] Setyaningsih W., Hidayah N., Saputro I.E., Palma M., García Barroso C. (2016). Profile of phenolic compounds in Indonesian rice (*Oryza sativa*) varieties throughout post-harvest practices. J. Food Compos. Anal..

[44] ISO/IEC 17025:2017 General Requirements for the Competence of Testing and Calibration Laboratories. http://faostat3.fao.org/browse/rankings/countries_by_commodity/E.

[45] (2005). ICH Topic Q2 (R1) Validation of Analytical Procedures: Text and Methodology. https://database.ich.org/sites/default/files/Q2_R1__Guideline.pdf.

[46] Zakiyah N.M., Handoyo T., Kim K.M. (2019). Genetic Diversity Analysis of Indonesian Aromatic Rice Varieties (*Oryza sativa* L.) Using RAPD. J. Crop. Sci. Biotechnol..

[47] Giraud G. (2013). The World Market of Fragrant Rice, Main Issues and Perspectives. Int. Food Agribus. Manag. Rev..

[48] Funsueb S., Krongchai C., Mahatheeranont S., Kittiwachana S. (2016). Prediction of 2-acetyl-1-pyrroline content in grains of Thai Jasmine rice based on planting condition, plant growth and yield component data using chemometrics. Chemom. Intell. Lab. Syst..

[49] Wakte K.V., Thengane R.J., Jawali N., Nadaf A.B. (2010). Optimization of HS-SPME conditions for quantification of 2-acetyl-1-pyrroline and study of other volatiles in Pandanus amaryllifolius Roxb. Food Chem..

