# Supplementary material for: Key-Marker Volatile Compounds in Aromatic Rice (Oryza sativa) Grains: An HS-SPME Extraction Method Combined with GC×GC-TOFMS
Source: Molecules. 2019 Nov 18;24(22):4180. doi: 10.3390/molecules24224180 (PMC6891657; doi:10.3390/molecules24224180)
Supplement: Supplementary file 1 [file molecules-24-04180-s001.pdf]

Supplementary Materials

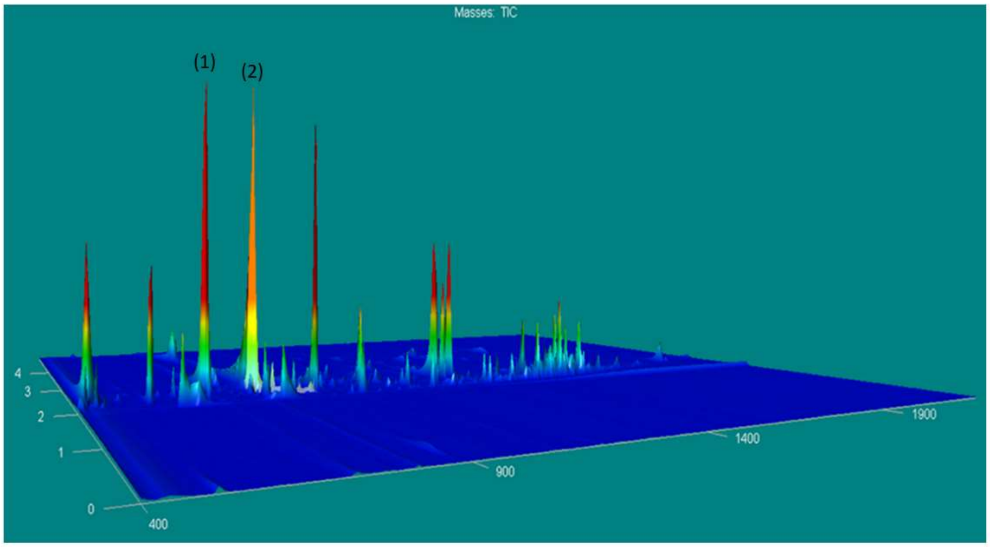

Figure S1. 2-acetyl-1-pyrroline (1) and internal standard 2,4,6-trimethylpyridine (2) in Pandan Leaf.

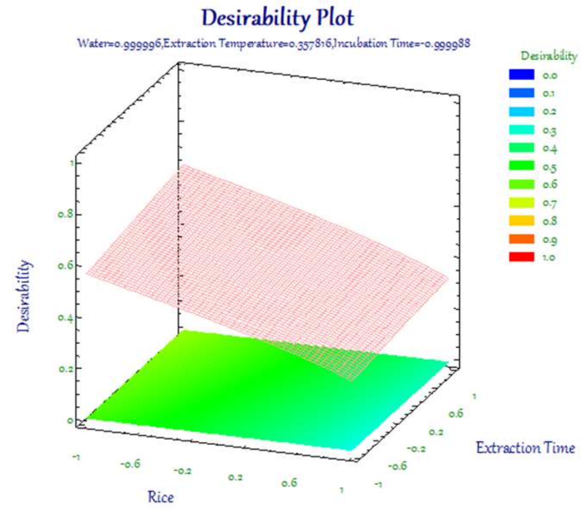

Figure S2. Response surface plots showing the effects of variables (x_1, sample amount; x_5, adsorption time) on the extraction yield.

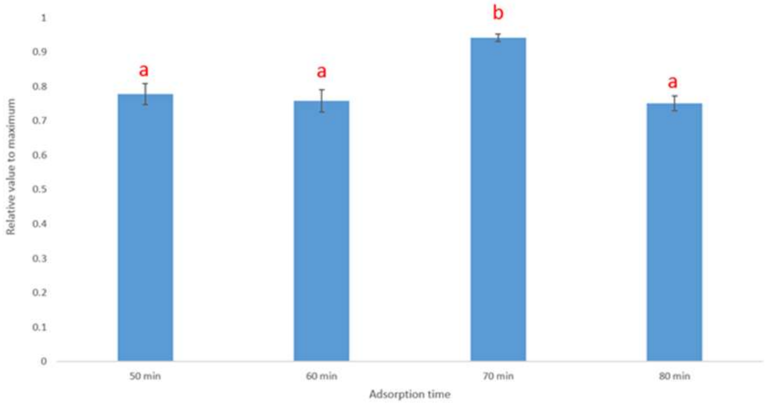

Figure S3. Relative amount of extracted compounds in different adsorption times.
